# New Insights into the Application of Biocompatible (Un)Modified TiO_2_ and TiO_2_-ZrO_2_ Oxide Fillers in Light-Curing Materials

**DOI:** 10.3390/ma17122908

**Published:** 2024-06-14

**Authors:** Katarzyna Siwińska-Ciesielczyk, Angelika Andrzejczak, Teofil Jesionowski, Łukasz Gierz, Agnieszka Marcinkowska, Mariola Robakowska

**Affiliations:** 1 Institute of Chemical Technology and Engineering, Faculty of Chemical Technology, Poznan University of Technology, Berdychowo 4, PL-60965 Poznan, Poland; katarzyna.siwinska-ciesielczyk@put.poznan.pl (K.S.-C.); andrzejczak194@wp.pl (A.A.); teofil.jesionowski@put.poznan.pl (T.J.); agnieszka.marcinkowska@put.poznan.pl (A.M.); 2 Institute of Machine Design, Faculty of Mechanical Engineering, Poznan University of Technology, Piotrowo 3, PL-60965 Poznan, Poland

**Keywords:** inorganic oxide fillers, kinetics of photocuring, UV-curable composite, wettability

## Abstract

A novel UV-light-curable poly(ethylene glycol) diacrylate matrix composite material with unmodified and methacryloxyl-grafted TiO_2_ and TiO_2_-ZrO_2_ systems was developed and tested as a potential coating material for medical components. The main goal of the research was to evaluate how the addition of (un)modified inorganic oxide fillers affects the properties of the composition (viscosity, UV/Vis spectra), the kinetics of photocuring (photo-DSC), and the morphological (SEM), physicochemical, and thermal properties (DSC, TGA) of the resulting composites. The applied filler functionalization process decreased their polarity and changed their size, BET surface area, and pore volume, which influenced the viscosity and kinetics of the photocurable system. In addition, the addition of synthesized fillers reduced the polymer’s glass transition temperature and increased its thermal stability. It was also observed that additional UV irradiation of the tested composite changed its surface, resulting in hydrophobic properties (with the addition of 7 wt.% filler, an increase in the contact angle by more than 45% was observed).

## 1. Introduction

The photoinitiated polymerization of multifunctional monomers provides an easy method to develop highly cross-linked polymer networks with diverse physicochemical properties that have broad industrial applications, for example, in the production of adhesives, paints, coatings, inks, varnishes, bone glues, or dental restorative materials [1,2,3]. The main advantage of UV curing is the rapid cure rates and high conversion that can be obtained under appropriate intense irradiation [4,5,6]. This technique is usually performed at room temperature and without the need for a solvent, making the UV-curing process an environmentally friendly and energy-efficient alternative to the traditional thermal curing of solvent-based polymer formulations.

Over the years, this technique has been used to obtain not only pure polymer matrices, but also polymer composites, where several fillers have been introduced into photocurable formulations to improve the specific properties of materials required for various applications [7,8,9]. Polymer composites have a combination of embedded inorganic materials and the polymer matrix, and therefore, these products can show novel properties and functions (e.g., optical, mechanical, thermal, catalytic, or conductive) [10,11,12]. A wide range of different materials are used as fillers in UV-curable products, including silica, alumina, titania, calcium carbonate, nickel(II) oxide, layered silicate, boron nitride, graphene derivatives, etc. [13]. Among the various inorganic materials, TiO_2_ is nontoxic, cheap, and chemically inert, has a high refractive index (~2.5 and ~2.7 for the anatase and rutile forms, respectively), and good photocatalytic properties. Therefore, it is also applied in the pharmaceutical industry and in food processing (as an antiseptic material, antibacterial compositions, and photoactive material that degrades various organic contaminants), in photocatalysis (photocatalytic conversion in solar cells), and as a filler of polymer nanocomposites in optical and electronic devices, sensors, biosensors, etc. [14,15,16]. Another inorganic filler that may have potential application in the fabrication of photocurable polymer composites is zirconia (ZrO_2_), which offers excellent hardness, mechanical strength, chemical and thermal stability, a high melting point, low electrical conductivity, wear resistance, and biocompatibility [17,18,19,20]. All of these properties favor its use in transparent optical devices, fuel cells, catalysts, sensors, microelectronics, ceramics, and biomaterials [20]. Nanooxides are a great alternative, e.g., as UV photodetectors based on ZnO nanowires [21] or intelligent emitters of thermally controlled radiation based on a layered structure formed by a phase change material (vanadium dioxide) [22].

Among various types of photoinitiators, those based on inorganic materials are the group of compounds least commonly used to initiate the photopolymerization process. It is well known that the addition of TiO_2_ to a photocurable composition causes light scattering (due to the high refractive index) and can have a degrading effect on polymer matrices. However, some authors have reported that TiO_2_ may catalyze the photopolymerization reaction of acrylate monomers, but different results have been obtained with respect to the acceleration [23,24] or deceleration of polymerization [25] due to the addition of TiO_2_. Ingrosso et al. [24] report that the addition of TiO_2_ increases the curing rate, increases the density, and reduces the inhibitory effect of oxygen on photopolymerization. This result is explained on the basis of the photoactivity of TiO_2_, which acts as a free radical donor photocatalyst in the photopolymerization reaction, ultimately proving to be more effective than a widely used commercial photoinitiator. A similar observation, namely the strong absorption of UV radiation by TiO_2_ nanoparticles, can be explained by the retarding effect of the addition of this filler on the photopolymerization rate of the epoxy acrylate system [25]. On the other hand, Damm et al. [26] note that the photopolymerization rate of ethoxylated trisacrylate with the addition of TiO_2_ strongly depends on the nature of its particles. They report that amorphous TiO_2_ is unable to initiate acrylate photopolymerization, while the introduction of pure anatase particles contributes to an increase in the polymerization rate with an increase in the average diameter of the particles and a decrease in their BET surface area [26]. Furthermore, it should be noted that the synthesis of mixed oxide systems composed of TiO_2_ and ZrO_2_ can reduce the photocatalytic activity of TiO_2_ due to the introduction of ZrO_2_ [27].

In dental applications, materials containing metal ions can exist in various forms, such as metal oxides (e.g., ZnO, TiO_2_, and Al_2_O_3_ [28]), solid metal nanoparticles (e.g., Ag nanoparticles), or as composite materials with layers of different metals. Dental materials are light cured, which is why the interaction between the filler and UV/Vis rays is so important. The properties of TiO_2_ are closely related to UV/Vis light, for example, a TiO_2_ surface under UV-light irradiation shows self-cleaning and antifogging characteristics [29]. According to Wang et al. [30,31,32], the surface of a TiO_2_ particle has light-induced superamphiphilicity (photoinduced hydrophilicity), which means that, during prolonged exposure to ultraviolet light, the surface of TiO_2_ is completely wetted with the liquids of water and oil (contact angle = 0°). The switchable wettability can be observed on both anatase and rutile TiO_2_ forms [29].

One of the main goals of the research was to check the possibility of using synthesized TiO_2_-based materials not only as active fillers, but also as initiators of the photopolymerization of the methacrylic monomer. As part of the investigation, inorganic oxide fillers (TiO_2_ and TiO_2_-ZrO_2_) were synthesized and then modified (with 3-(trimethoxysilyl)propyl methacrylate) and used in a light-cured polymer matrix. TiO_2_ particles have many favorable properties, such as chemical stability, biocompatibility, and low toxicity to human tissues [33], but little data are available in the literature on the use of TiO_2_ and TiO_2_-ZrO_2_ for photocurable dental fillings and glass ionomer cements [33]. However, there is a complete lack of reports on the use of TiO_2_-ZrO_2_ as a photoinitiating system and the behavior under the influence of light is different from the literature data.

In this paper, the synthesized inorganic materials were subjected to detailed physicochemical analysis to determine the impacts of synthesis and modification conditions on their morphology, crystalline, and textural structure. Moreover, the influence of the addition of inorganic fillers on the physicochemical, kinetic, and thermal properties of the polymer matrix was determined. The results provide important information enabling a good understanding of such photocurable composites and their potential in dental and biomedical applications.

## 2. Materials and Methods

### 2.1. Chemicals and Materials

Titanium(IV) isopropoxide (TTIP, 97%), zirconium(IV) propoxide (TZIP, solution 70 wt.% in 1-propanol), hexadecylamine (HDA, 99%), 3-(trimethoxysilyl)propyl methacrylate (silane A-174, 98%), poly(ethylene glycol) diacrylate (PEGDA, 99%, M_n_ = 575), and 2,2-dimethoxy-2-phenylacetophenone (DMPA, 99%) were acquired from Sigma-Aldrich (St. Louis, MO, USA). Potassium chloride (KCl, 99.9%), ethanol (EtOH, 99.6%) and methanol (MeOH, 99.8%) were purchased from Chempur (Piekary Śląskie, Poland). Deionized water was used in all experiments. All reagents were used without any further purification.

### 2.2. Fabrication of Inorganic Oxide Fillers

**Figure 1:**
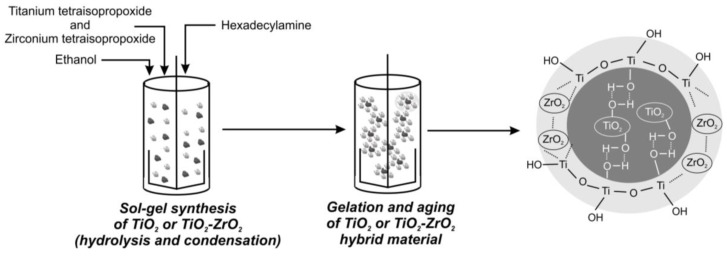
Synthesis of TiO_2_ or TiO_2_-ZrO_2_ inorganic fillers via the sol-gel method.

The fabrication of inorganic oxide fillers (TiO_2_—sample T; and TiO_2_-ZrO_2_—sample TZ) was carried out using a sol-gel method assisted by a calcination process at 500 °C. Firstly, an appropriate volume of solvent (ethanol) was introduced into a three-neck flask equipped with a T25 Basic high-speed stirrer (Ika Werke GmbH, Staufen im Breisgau, Germany). In the next step, an appropriate mass of shape controller (hexadecylamine) was added to the solvent. When the shape controller was dissolved, 0.1 M KCl was introduced into the reaction mixture using an automatic pipette. To the resulting mixture, organic precursors of inorganic oxides (TiO_2_ and ZrO_2_) were added using an ISM833A peristaltic pump (ISMATEC, Wertheim, Germany) at a constant rate of 1 cm^3^/min. In the synthesis of ‘pure’ TiO_2_, the precursor of ZrO_2_ was not introduced into the reaction mixture. The reaction mixture was homogenized at 700 rpm. To allow the hydrolysis and condensation reactions of organic precursors of inorganic oxides to take place, after their addition, the mixture was stirred for 1 h. The resulting suspensions were allowed to age for 48 h at room temperature. The obtained precipitates were dried at 60 °C for 8 h. After drying, they were washed four times with water and once with ethanol, and were again dried for 4 h at 60 °C. In the next step, the samples were calcined at 500 °C for 2 h using a Nabertherm Controller P320 oven (heating rate 5 °C/min). To obtain the final products, samples were ground and sifted through a sieve with a mesh diameter of 80 µm after the calcination process. The schematic methodology for the synthesis of TiO_2_ or TiO_2_-ZrO_2_ inorganic oxide materials is presented in Figure 1.

### 2.3. Surface Modification of Inorganic Oxide Systems

To increase the adhesion (interaction) between the inorganic oxide material (TiO_2_ or TiO_2_-ZrO_2_) and the polymer, the fabricated oxide materials were subjected to surface functionalization using the solvent evaporation method (classified as a dry technique). First, to a reactor equipped with a magnetic stirrer, an appropriate volume of solvent (methanol/water—4/1 *v*/*v*) was introduced, and a silane coupling agent (3-(trimethoxysilyl)propyl methacrylate) was added in an amount of 10 parts by mass of TiO_2_ or TiO_2_-ZrO_2_ (100.0 g). The silane coupling agent A-174 was hydrolyzed and condensed in the reactive mixture. Next, an appropriate mass of inorganic oxide material was introduced into the mixture. The resulting dispersion was then stirred for 1 h to homogenize the sample with the solution of the modifying agent. Finally, the solvent was distilled off, and the silane-grafted samples were dried at 105 °C for 2 h. The final step was to grind and sieve the obtained products (samples T_M_ and TZ_M_) through a sieve with a mesh diameter of 80 µm.

### 2.4. Preparation of Light-Curing Compositions

Poly(ethylene glycol) diacrylate (PEGDA) was used as the monomer. Monomer/inorganic oxide material (unmodified and modified TiO_2_ or TiO_2_-ZrO_2_) mixtures containing 0, 3, 5, or 7 wt.% of the filler and 0.5 wt.% of the photoinitiator (DMPA) were homogenized. The homogenization process was carried out using a mechanical shaker (15 h, 3000 rpm) and an ultrasonic bath (24 h, 50 kHz, 310 W) for 10 h. Systems without a photoinitiator were also used for kinetic studies.

**Figure 2:**
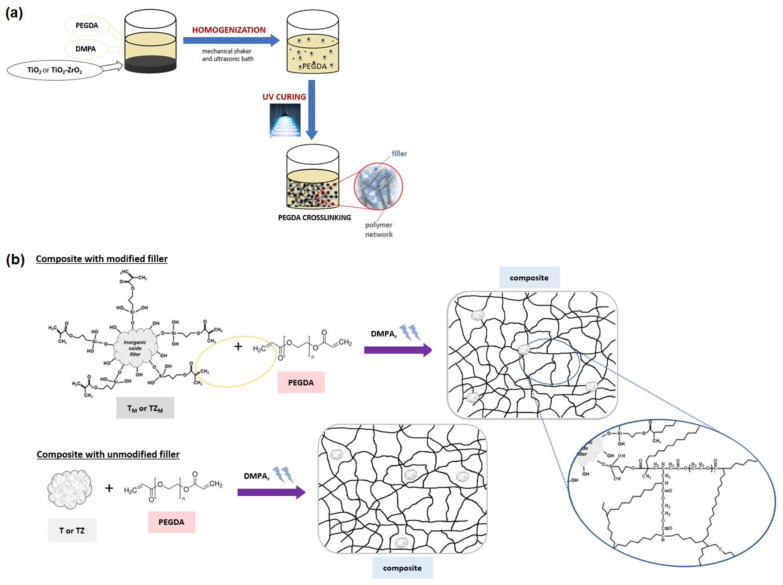
(**a**) Composite preparation scheme and (**b**) the structure of the composite with modified and unmodified fillers incorporated into the network structure.

The preparation of the composites by photopolymerization as well as the structure of the composites with fillers (modified by methacryloxy groups and unmodified) incorporated into the network structure are shown schematically in Figure 2.

### 2.5. Characterization of Obtained Inorganic Oxide Fillers

To evaluate the dispersive properties of the fabricated inorganic oxide fillers, the non-invasive backscattering (NIBS) method was applied using a Zetasizer Nano ZS apparatus (Malvern Instruments Ltd., Worcester, UK). The synthesized sample (0.01 g) was dispersed in 25 cm^3^ of propan-2-ol, sonicated for 15 min, and then placed in a cuvette for analysis.

Visualization of the surface morphology of the obtained inorganic fillers and hybrid materials was performed by scanning electron microscopy (SEM) using a Tescan MIRA3 apparatus (Princeton Gamma-Tech, Princeton, NJ, USA).

To verify the crystalline structure of the synthesized inorganic fillers, X-ray powder diffraction analysis (XRD) was performed using a TUR-M62 diffractometer (Carl Zeiss, Jena, Germany; Cu Kα radiation, α = 1.5418 Å, Ni filtered, Δ2θ = 0.04°, 2θ = 10–80°).

The textural properties of the inorganic oxide fillers, including surface area (*A_BET_*), average pore diameter (*S_p_*), and total pore volume (*V_p_*), were determined by low-temperature N_2_ sorption at −196 °C in an ASAP 2020 physisorption analyzer (Micromeritics Instrument Co., Norcross, CA, USA). The surface area was calculated from the adsorption isotherm in the relative pressure range of *p*/*p*_0_ = 0.05–0.3 based on the Brunauer–Emmett–Teller (BET) model. The pore size distribution and total volume of pores were determined from the desorption isotherm by the Barrett–Joyner–Halenda (BJH) method, using the Halsey equation. Before measurement, the synthesized materials were degassed at 120 °C for 4 h.

To confirm the effectiveness of the modification process, the fabricated inorganic fillers were subjected to elemental analysis using a Vario EL Cube apparatus from Elementar Analysensysteme GmbH (Langenselbold, Germany).

To identify the characteristic functional groups present in the materials, Fourier transform infrared (FTIR) spectroscopy was performed. FTIR spectra were obtained in a wavenumber range of 4000 to 400 cm^−1^ using a Vertex 70 apparatus from Bruker (Karlsruhe, Germany). The samples were prepared by mixing with KBr and pressing into small tablets.

### 2.6. Characterization of Light-Curing Compositions and Polymer Composites

The viscosity of the monomer/inorganic oxide filler compositions was measured in a shear rate range of 0–200 rpm with a Brookfield Digital Viscometer model DV-II (cone-plate geometry) at 20 °C (the temperature of the polymerization process).

The photopolymerization rate profiles were monitored by differential scanning calorimetry (DSC) under isothermal conditions at 20 ± 0.01 °C, in a high-purity argon atmosphere (<0.0005% O_2_) using a Pyris 6 instrument (Perkin-Elmer, Waltham, MA, USA) equipped with a lid specially designed for photochemical measurements. A sample weighing 2 mg was polymerized in an open aluminum crucible with a diameter of 6.6 mm. Polymerization was initiated by UV light from an LED LC-L1 Hamamatsu lamp (λ_max_ = 365 nm; light intensity of the sample: 2.75 mW/cm^2^). Inert gas (argon) was passed at a rate of 50 cm^3^/min through the apparatus chamber for 5 min before and during the whole measurement. The reproducibility of the kinetic results was approximately ±3%. For the polymerization computations, the heat was taken to be 78 kJ/mol per double bond [34].

Polymer and polymer composites (dimensions: diameter of 12 mm and thickness of 1 mm) were obtained by photocuring the prepared compositions in a metal mold. The samples were irradiated through PET foils for 3 min on both sides of the mold in a Flood Lamp 500, DYMAX (I_0_ = 105 mW/cm^2^) using its full light spectrum (320–395 nm).

The thermal resistance of the composites was investigated with a Tarsus TG 209 F3 thermogravimetric analyzer (Netzsch-Geratebau GmbH, Selb, Germany). Samples of 10 mg were heated in Al_2_O_3_ crucibles in the temperature range of 35–600 °C at a scan rate of 10 °C/min under a nitrogen atmosphere (flow rate of purge gas: 10 cm^3^/min; flow rate of protective gas: 20 cm^3^/min).

The glass transition temperature, *T_g_*, was measured with a DSC1 instrument (Mettler Toledo GmbH, Schwerzenbach, Switzerland) under a nitrogen atmosphere. Samples of polymers or composites weighing 5 mg were scanned at a heating/cooling rate of 20 °C/min in a temperature range from −80 to 100 °C. The *T_g_* values were evaluated from thermograms obtained from the first and second runs of the heating mode of two separate DSC measurements.

The absorption spectrum for each filler was measured using a Jasco UV-530 spectrophotometer (Tokyo, Japan). All spectroscopic measurements were carried out in a measuring range of 200 to 800 nm, in a quartz cuvette with a light-path length of 1 mm at room temperature.

Fourier transform infrared (FTIR) spectra of the products were obtained in the range of 4000–600 cm^−1^ using an attenuated total reflectance (ATR) accessory (Nicolet 5700 equipped with a ZnSe crystal ATR unit, Thermo Fisher Scientific Inc., Waltham, MA, USA, and Bruker Tensor 27 equipped with a SPECAC Golden Gate diamond ATR accessory, Bruker Optik GmbH, Ettlingen, Germany). Spectra were recorded with a resolution of 4 cm^−1^ and with 64 scans.

The contact angle of the samples was measured using an OCA 15EC contact angle goniometer (DataPhysics Instruments GmbH, Filderstadt, Germany) at room temperature (approximately 25 °C) and with an accuracy of ±0.01 mN/m. Water droplets of a volume of 0.2 μL were deposited at ten random locations on the surface of the composite. The images of the droplets were recorded and analyzed using SCA20. v 2017 shape analysis software (DataPhysics Instruments GmbH, Filderstadt, Germany). The water contact angle of the samples was measured before and after additional UV-light irradiation in the Flood Lamp 500, DYMAX, Torrington, CT, USA (I = 105 mW/cm^2^).

## 3. Results and Discussion

### 3.1. Physicochemical Characterization of Inorganic Oxide Fillers

The physicochemical analysis of the produced fillers began with the determination of the particle size distribution (Table 1), which is one of the key parameters affecting the quality of the obtained polymeric materials and the ability to initiate photopolymerization reactions. The synthesized TiO_2_ (sample T) contained particles in the diameter range of 164–531 nm. In turn, dispersive analysis of TiO_2_ subjected to surface modification with a selected silane coupling agent (sample T_M_) showed the presence of particles in the diameter range of 459–1110 nm. In the case of sample T, the dominant contribution (22.7%) came from particles 295 nm in diameter, while for T_M_, the maximum volume contribution (28.2%) was from particles with a diameter of 712 nm. The polydispersity indices of these samples were 0.382 and 0.225, respectively. Dispersive analysis of samples TZ and TZ_M_ showed the presence of particles in the diameter ranges of 342–615 nm and 459–825 nm, respectively, with the maximum volume contributions of particles with diameters of 459 nm (43.0%) and 615 nm (38.8%), respectively. Detailed dispersive analysis of the obtained materials showed that the addition of a second component, as well as the surface hydrophilization of the inorganic oxide fillers, caused a slight shift in the particle diameter toward higher values.

**Figure 3:**
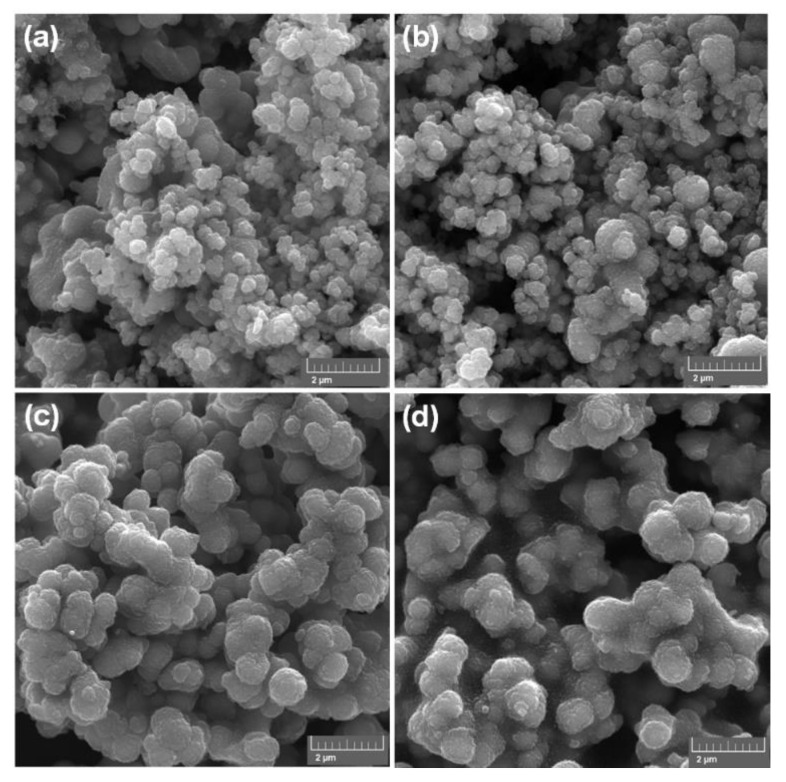
Morphology of inorganic oxide fillers: (**a**) T, (**b**) T_M_, (**c**) TZ, and (**d**) TZ_M_.

The next step in the physicochemical characterization involved the evaluation of the effect of the surface modification with a silane coupling agent on the morphology and microstructure of the obtained materials (Figure 3). SEM microphotographs of all fabricated fillers (unmodified and modified inorganic oxide fillers) confirmed the presence of particles with diameters corresponding to those indicated in the dispersive analysis, ovoid in shape, having a visible tendency to form aggregates.

**Figure 4:**
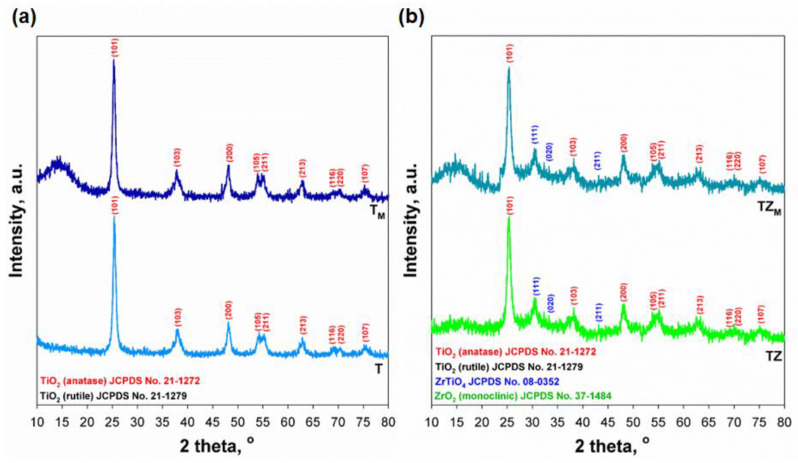
Crystalline structure of: (**a**) samples T and T_M_, and (**b**) samples TZ and TZ_M_.

The XRD patterns of the unmodified and modified inorganic oxide fillers are presented in Figure 4. For samples T and T_M_, the diffraction peaks at 2θ = 25.28, 38.58, 48.05, 53.89, 55.06, 62.69, 68.76, 70.31, and 75.03, which correspond to the planes (1 0 1), (1 0 3), (2 0 0), (1 0 5), (2 1 1), (2 1 3), (1 1 6), (2 2 0), and (1 0 7), can be assigned to the anatase structure of TiO_2_ (JCPDS no. 21-1272). For these samples, there were no visible diffraction peaks indicating the presence of rutile; the anatase phase content in these samples was 100% (Table 2). The diffractograms for pure TiO_2_-ZrO_2_ (sample TZ) and the same material subjected to surface modification with the selected modifier (sample TZ_M_) show not only diffraction maxima originating from the anatase structure of TiO_2_, but also peaks related to the presence of the zirconium titanate (ZrTiO_4_) structure (JCPDS no. 34-0415). The reflections observed at 2θ = 30.44, 32.61, and 44.93 can be indexed respectively to the (1 1 1), (0 2 0), and (2 1 1) planes of ZrTiO_4_. In these samples, TiO_2_ is the dominant phase with a content of 79.2%, while the content of ZrTiO_4_ is 20.8%. The presence of the crystalline structure of ZrTiO_4_ confirms that the proposed synthesis method is effective and that Zr atoms have partially replaced the Ti atoms, leading to a mixed oxide system [35,36]. X-ray analysis of the fabricated materials shows that the surface functionalization process does not change the crystalline structure of the inorganic oxide fillers. Moreover, the interaction between the inorganic filler and silane occurred on the surface. A similar observation was made by Aydinoğlu [37].

**Figure 5:**
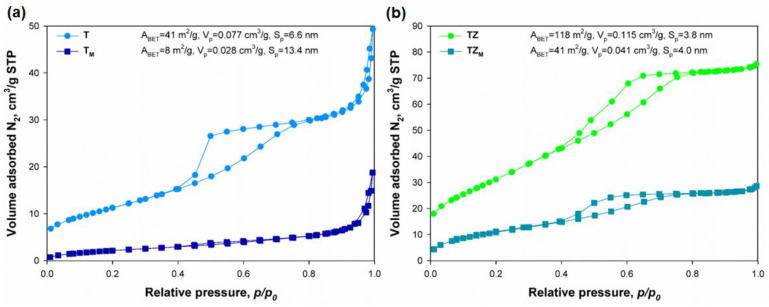
N_2_ adsorption/desorption isotherms of: (**a**) T and T_M_, and (**b**) TZ and TZ_M_ inorganic oxide fillers.

Low-temperature N_2_ sorption analysis was performed to evaluate the textural properties of the fabricated inorganic oxide fillers, including surface area (*A_BET_*), total pore volume (*V_p_*), and average pore diameter (*S_p_*). The N_2_ sorption isotherms of all fillers (see Figure 5) can be classified as type IV with a type-H3 hysteresis loop, according to the IUPAC classification [38]. The values of porous structure parameters obtained for unmodified and modified TiO_2_ (samples T and T_M_; see Figure 5a) prove that the surface modification process with the selected silane coupling agent significantly reduced the BET surface area of the inorganic material filler. Sample T (TiO_2_) had a BET surface area of 41 m^2^/g, while the BET surface area of the modified TiO_2_ (T_M_) was 8 m^2^/g. The average pore diameter (*S_p_*) values for these materials were 6.6 nm (sample T) and 13.4 nm (sample T_M_), and the respective total pore volume (*V_p_*) values were 0.077 cm^3^/g and 0.028 cm^3^/g. The TiO_2_-ZrO_2_ oxide system (TZ sample) had the highest value of *A_BET_* (118 m^2^/g) among all of the fabricated fillers. For this sample, the value of *S_p_* was 3.8 nm and *V_p_* was 0.115 cm^3^/g. For the TZ_M_ sample (modified TiO_2_-ZrO_2_ material), the BET surface area was three-times smaller than that of the unmodified TiO_2_-ZrO_2_ sample, at 41 m^2^/g (Figure 5b). For this sample, the average pore diameter (*S_p_*) was 4.0 nm, and the total pore volume (*V_p_*) was 0.041 cm^3^/g. The interpretation of the low-temperature N_2_ sorption results clearly confirms that the process of surface modification of the inorganic fillers with the selected silane coupling agent A-174 contributed to a decrease in the BET surface area and total pore volume, and an increase in the average pore diameter. The reduction in the BET surface area for silane-modified samples is a consequence of the fact that active centers (hydroxyl groups) on the surfaces of TiO_2_ and TiO_2_-ZrO_2_ are blocked by the modifier. This result may confirm that a chemical reaction occurred between the modifier and the surface hydroxyl groups of the inorganic oxide fillers. Similar observations have been reported by other researchers [39,40,41].

In the next step of the physicochemical characterization of the fabricated inorganic oxide fillers, to confirm the effectiveness of the modification process, the fillers were evaluated by elemental analysis. The results (Table 3) show that the content of carbon and hydrogen increased after the modification of the filler surface with 3-(trimethoxysilyl)propyl methacrylate. For the modified samples, the degrees of coverage (P) with the selected modifier A-174 were additionally calculated from the Berendsen and de Golan Equation (1) [42], using the results of the elemental and BET analysis:

(1) $$P=\frac{10^{6}\cdot C}{[1200\cdot N_{C}-C\left(M-1\right)]\cdot A}$$

where C is the carbon content of the sample, N_C_ is the number of carbon atoms in the attached molecule, M is the molar mass of the attached compound, and A is the BET surface area of the unmodified oxide material. The values of the degree of coverage of inorganic fillers with modifier were 5.5 and 1.8 μmol/m^2^ for samples T_M_ and TZ_M_, respectively.

**Figure 6:**
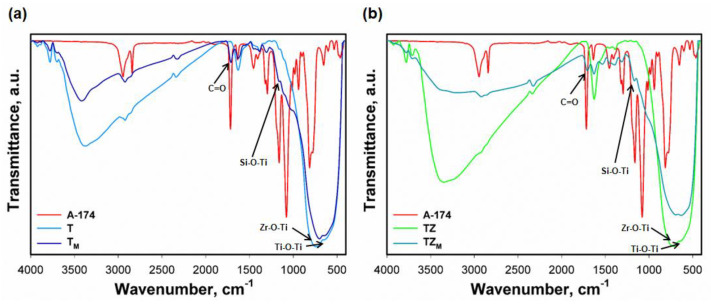
FTIR spectra of fabricated materials.

The chemical structure of the materials was investigated using FTIR spectroscopy. The analysis made it possible to confirm whether the process of the surface modification of fillers allowed the introduction of suitable functional groups on the surface of the produced materials to increase the interaction between the inorganic filler and the polymeric material (Figure 6a,b and Table 4). The FTIR spectrum of 3-(trimethoxysilyl) propyl methacrylate shows characteristic absorption bands at 830 cm^−1^, 910–890 cm^−1^, 1080 cm^−1^, 1087 cm^−1^, 1160 cm^−1^, 1410 cm^−1^, 2830 cm^−1^, 2950 cm^−1^, and 2973 cm^−1^, which are attributed to Si-CH_2_, Si-OH, Si-O, Si-O-C, Si-O-CH_3_, C-H, -CH_2_- (asymmetric), and -CH_2_- (symmetric) groups [37,43,44]. For samples T and TZ, the absorption bands at 714 cm^−1^ and 640 cm^−1^ correspond to symmetric stretching vibrations of Ti-O-Ti groups [36]. The bands at wavenumbers 742 cm^−1^ and 721 cm^−1^ indicate the presence of Zr-O and Ti-O-Zr groups, respectively [36]. These samples also produce an absorption band in the range of 3700–3200 cm^−1^, corresponding to hydroxyl groups (-OH). In the case of T_M_ and TZ_M_ samples, the decrease in the intensity of the bands at 3700–3200 cm^−1^ and 1640 cm^−1^ may confirm the effective modification of the inorganic fillers (TiO_2_ and TiO_2_-ZrO_2_) with 3-(trimethoxysilyl)propyl methacrylate. It is important to note that the modified samples produce a new band at a wavenumber of 1720 cm^−1^, characteristic of the C=O group, which indicates a successful modification process. In addition, in the case of the modified samples (T_M_ and TZ_M_), there is a noticeable increase in band intensity in the range of 1170–1080 cm^−1^, which indicates the formation of Si-O-Ti bonds.

**Figure 7:**
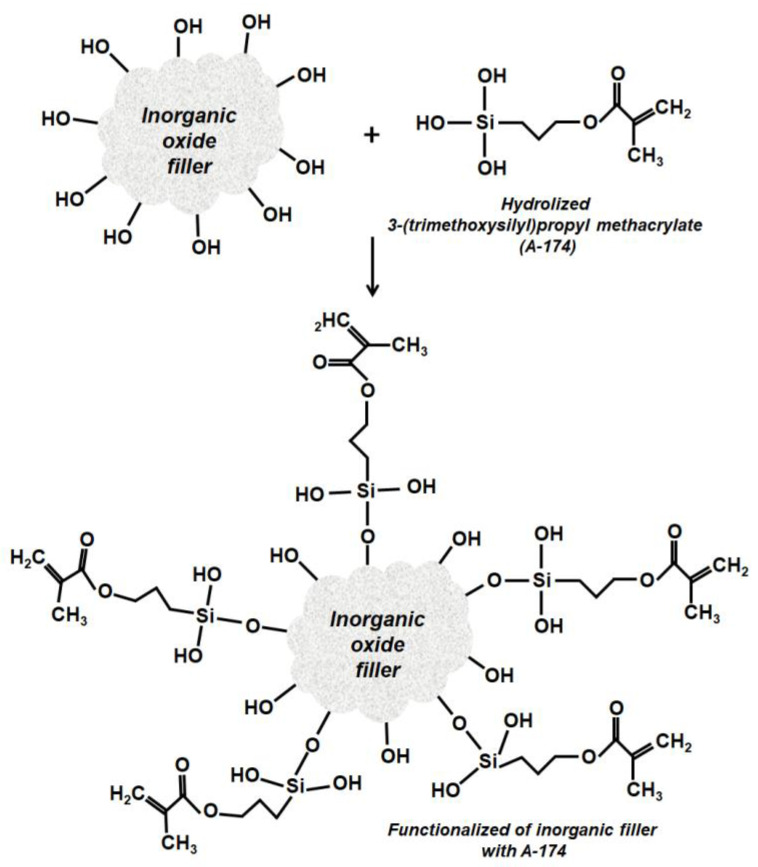
Proposed mechanism of reaction between 3-(trimethoxysilyl)propyl methacrylate (A-174) and the surface of the inorganic oxide fillers.

Based on the results of the analysis, a mechanism was proposed for the interaction between the inorganic filler and the silane coupling agent (Figure 7).

### 3.2. Characterization of Monomer/Inorganic Oxide Filler Compositions

#### 3.2.1. Viscosity

**Figure 8:**
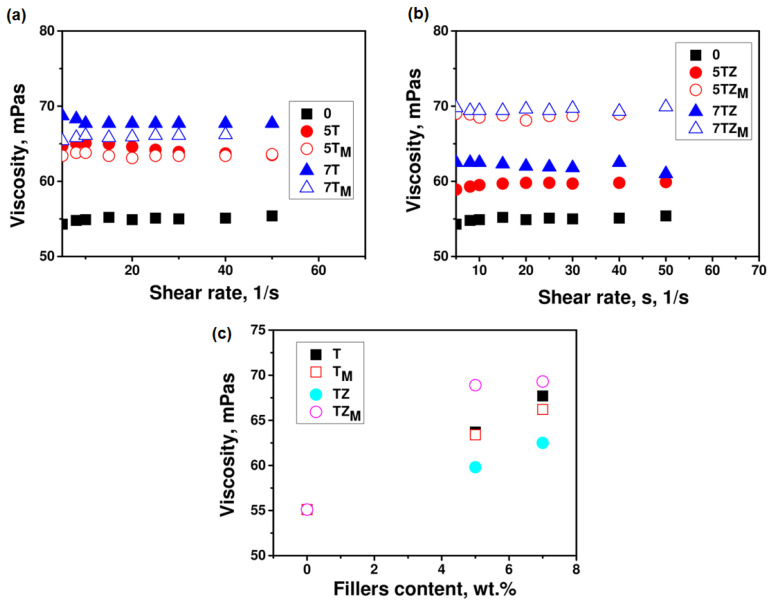
Viscosity as a function of the shear rate for compositions containing PEGDA and (**a**) T/T_M_ and (**b**) TZ/TZ_M_ fillers, and (**c**) as a function of filler content at the shear rate of 40 1/s.

The viscosity of a UV-curable formulation is a very important parameter that affects the UV reactivity of the system and the polymerization rate. Figure 8 shows the viscosity of the monomer/filler dispersions as a function of the shear rate and the content and type of the filler. Measurements were taken at 20 °C, which was the temperature used for the kinetic studies and the preparation of the composites. The introduction of inorganic oxide fillers to the acrylate monomer modified its rheological properties, with the effect depending on the nature of the filler (modification, type, and amount of filler). The measured viscosity of the monomer without fillers presented Newtonian behavior with an apparent viscosity of 55 mPa·s, which is comparable to the data from the literature [45]. The results demonstrate that the addition of even a small amount of prepared inorganic oxide filler (unmodified or modified) increases the initial viscosity of the system. The addition of 5 wt.% of unmodified TiO_2_ and TiO_2_-ZrO_2_ caused an increase in the viscosity of the compositions, respectively, by ~15% and ~8.5%. The applied modifications of the initial TiO_2_ had different effects on the viscosity of the composition. This may result on the one hand from a change in the size of the filler particles, and on the other hand from a change in monomer–filler interactions. After the modification of TiO_2_ with ZrO_2_, a decrease in viscosity is observed. The modification of TiO_2_ with the silane coupling agent A-174 also reduced the viscosity of the composition, but the decrease was insignificant. On the other hand, the modification of the TZ filler with a silane coupling agent results in a significant increase in the viscosity of the composition, relative to the viscosities produced by the use of both unmodified TZ and T samples (TiO_2_-ZrO_2_ and TiO_2_ fillers).

The surface treatment of the fillers reduced their polarity, which also reduced particle–particle interactions and made the TiO_2_ and TiO_2_-ZrO_2_ surfaces more compatible with the polymer matrix. The viscosity of the compositions increased with filler content, but the increase was much stronger for systems containing silane-modified TiO_2_-ZrO_2_ (TZ_M_ sample). As mentioned above, the modification of T and TZ particles with silanes significantly changed their size, as well as their BET surface area and pore volume, which caused a variation in the viscosity of the initial system.

#### 3.2.2. Photocuring

**Figure 9:**
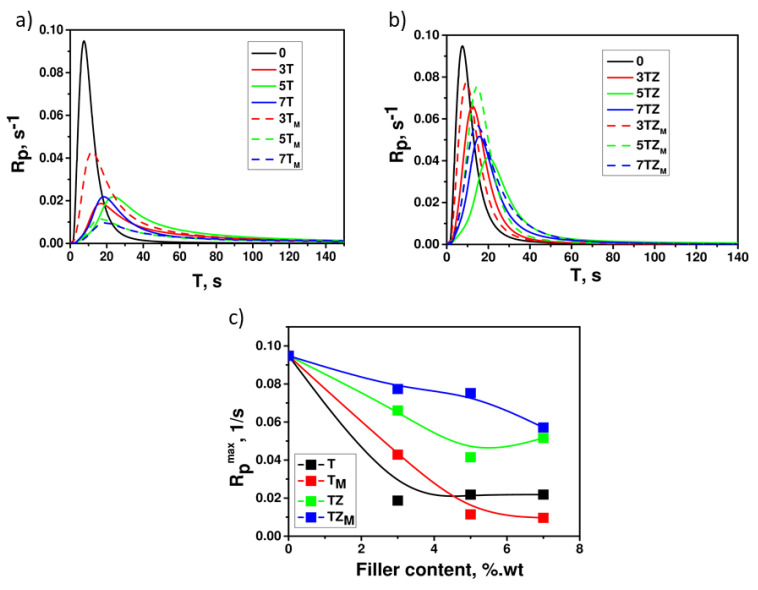
Kinetic curves of polymerization of PEGDA containing (**a**) T/T_M_; (**b**) TZ/TZ_M_ filters and (**c**) Maximum polymerization rate *R_p_^max^* depending on filler content. The numbers indicate filler content (in wt.%).

**Figure 10:**
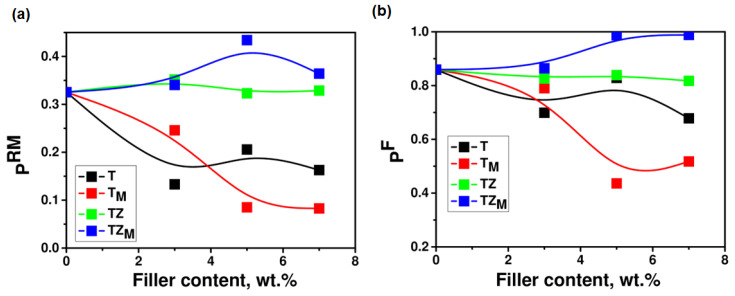
Double-bond conversion (**a**) at *R_p_^max^* (*p^RM^*) and (**b**) final (*p^F^*) as functions of the filler content in PEGDA containing samples T, T_M_, TZ, and TZ_M_.

In order to determine the effect of the addition of synthesized fillers on the reaction kinetics, the polymerization rate of systems containing PEGDA, various amounts of the fillers in the range of 0–7 wt.% (T, T_M_, TZ, and TZ_M_ samples), and a constant amount (0.5 wt.%) of the photoinitiator (DMPA) were investigated. Multifunctional monomers, which include PEGDA, are the basic group of compounds that undergo the photocuring process. The rate of photopolymerization of multifunctional monomers, *R_p_*, increases from the beginning of the reaction up to a certain point, reaching a maximum value, *R_p_^max^*. Once this value is reached, the reaction rate begins to decrease. This is influenced by factors such as a decrease in initiation efficiency, a decrease in monomer concentration, a limitation of the mobility of the polymer network, and the type of additive introduced. The acceleration process starts from the beginning of the polymerization reaction, because the diffusion limitations of termination occur from the beginning of the process, which is related to the formation of a polymer network and the attachment of radicals (active centers of polymerization) to it. The kinetic curves of the polymerization of PEGDA with and without the addition of filler have a shape characteristic of the polymerization of multifunctional monomers. The dependence of the polymerization rate, *R_p_*, on the irradiation time, *t*, for formulations with different filler contents shows an immediate onset of autoacceleration and the appearance of a maximum polymerization rate, *R_p_^max^* (Figure 9). The maximum polymerization rate, *R_p_^max^*, occurred for monomer PEGDA at 32% of the double-bond conversion (Figure 9b), and 10 s after the start of irradiation (Figure 10). The addition of fillers to the monomer affected the kinetics of the polymerization reaction, and a decrease in the polymerization rate, both initial and maximum, was observed. For a monomer containing a small amount of T or T_M_ filler, a sharp decrease in the reaction rate is observed; however, the addition of 7 wt.% of the filler does not cause a further decrease in the reaction rate (Figure 9a). In the case of the TZ and TZ_M_ fillers, the effect of slowing down PEGDA polymerization is smaller. Moreover, it was notable that the addition of 5 wt.% of modified TiO_2_ (T_M_ sample) caused a significant reduction in the polymerization rate compared to the unmodified system, while, in the case of modified TiO_2_-ZrO_2_ (TZ_M_ sample), an opposite effect was observed. Therefore, it may be concluded that the modification of TiO_2_ with ZrO_2_ had a positive effect on the curing kinetics. The addition of T and T_M_ fillers, apart from a rapid decrease in the PEGDA polymerization rate, also causes a significant decrease in the degree of double-bond conversions, both *p^Rm^* and *p^f^*, while in the case of the TZ filler, the decrease in conversion is negligible, and in the case of TZ_M_, even a slight increase in the degree of conversion is observed (Figure 9b,c). As can be seen in Figure 10, the conversion of double bonds *p^Rm^* and *p^f^* reached the highest values for the composition containing 5 wt.% of the TZ_M_ sample.

The addition of TiO_2_ particles to PEGDA, regardless of the amount of the filler, caused a decrease in the reaction rate compared to the pure monomer. Various reasons have been suggested for this. First, the uncertainty of the presence of crystalline TiO_2_ particles was excluded: as shown in Figure 4, samples T and T_M_ had a crystalline anatase structure. According to [26], amorphous TiO_2_ was not able to initiate the polymerization of the system. A second reason may be that the TiO_2_, despite consisting mainly of an anatase phase, had more UV-absorbing properties than polymerization-initiating properties and thus blocked access to radiation from the UV source. In addition, the light incident on the composition may be scattered by the filler particles, which may result in a decrease in the intensity of the light and thus in a decrease in the rate of initiation, in turn affecting the rate of polymerization. This would indicate differences in the dispersion of the filler (unmodified and modified T or TZ fillers) in the polymerizing medium. This possibility will be discussed later, in the section on the morphology of composites examined by SEM (Section 3.2.5).

**Figure 11:**
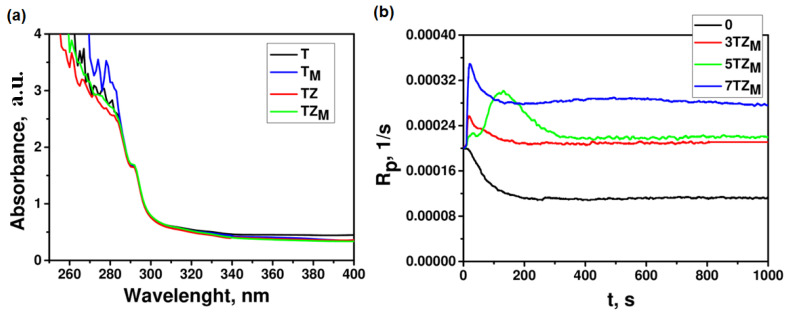
(**a**) UV-Vis spectra of fillers dispersed in ethanol; (**b**) kinetic curves of polymerization of PEGDA without photoinitiator DMPA containing TZ_M_ filler.

To investigate the effectiveness of UV-Vis-light absorption by the prepared fillers, the UV spectra of filler particles dispersed in ethanol were examined (Figure 11a). These spectra demonstrated that all prepared fillers absorbed UV/Vis light in a similar range, with noticeable absorption peaks appearing below 300 nm, while the photoinitiator absorbed at 366 nm.

An increase in the polymerization rate often correlates with an increase in the viscosity of the system, which suppresses termination by retarding the diffusion of macroradicals, causing an increase in the radical concentration and thus accelerating polymerization. This results in an inverse proportionality between *k_tb_* and system viscosity *η*: *k_tb_*~*η*^−1^. Thus, an increase in the initial viscosity of the formulation should result in faster polymerization. In our case, this was confirmed for the system containing the modified TZ_M_ sample; these compositions had the highest initial viscosity (Figure 8) and the highest polymerization rates (of the filled systems) and achieved the highest degrees of double-bond conversion (Figure 10b).

To verify whether the added fillers initiated photopolymerization, we investigated the photocuring kinetics of systems without the addition of a photoinitiator. Kinetic curves (Figure 9b) show the polymerization rates of PEGDA without photoinitiator containing 0–7 wt.% of TZ_M_ filler. The synthesized inorganic oxide materials exhibited insignificant photoinitiating capability for the polymerization reaction (the highest polymerization rate was recorded for a composition containing 7 wt.% of TZ_M_ filler). For the other systems, the polymerization rates of a composition without photoinitiator were negligible.

#### 3.2.3. Spectral Analysis

**Figure 12:**
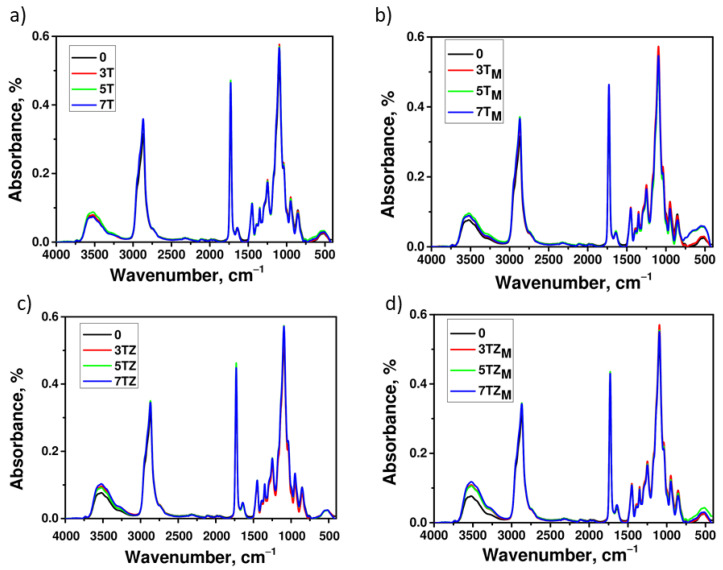
FTIR spectra recorded for the samples of polymer and polymer composite containing (**a**) T; (**b**) T_M_; (**c**) TZ; and (**d**) TZ_M_. The numbers indicate filler content (in wt.%).

The presence of polymer–filler interactions was investigated by observing shifts in the absorption bands of characteristic groups in FTIR spectra. As can be seen in Figure 12, few changes can be observed in the IR spectra of the composite materials. The broad absorption bands at 500–800 cm^−1^ correspond to Ti-O-Ti stretching. The spectra of the composites contain absorption bands at 3200–3600 cm^−1^, corresponding to vibrations of Ti-OH residue groups (and Zr-OH in samples TZ and TZ_M_), which increase slightly with the increasing content of filler in the material. Peaks at 2860–3000 cm^−1^ correspond to the asymmetric and symmetric stretching of C-H bonds, and bands at 1720–1725 cm^−1^ and 1110 cm^−1^ indicate the presence of C=O and O-C-O bonds (stretching) in the PEGDA molecule.

#### 3.2.4. Thermal Properties

**Figure 13:**
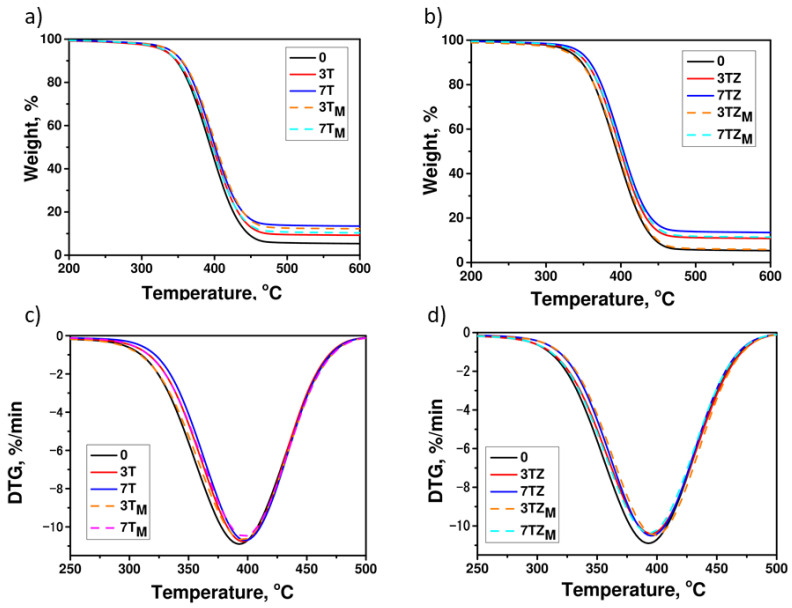
Thermal decomposition of polymer and polymer composite containing: (**a**) T/T_M_; (**b**) TZ/TZ_M_ and DTG curves: (**c**) T/T_M_; (**d**) TZ/TZ_M_. The numbers indicate filler content (in wt.%).

The TGA analysis provided information on the rate of decomposition of the samples during the heating process. Figure 13 and Table 5 show the TGA curves and thermal properties of the neat polymer and polymer composites, respectively. The decomposition of the investigated materials occurred in one stage, with the maximum rate of material decomposition occurring at a temperature around 392–400 °C, which may depend on the decomposition of the polymer material (rupture of -C-C- chain bonds). According to the data from the literature [46], the anatase phase of TiO_2_ has superior thermal stability from 288 °C to 800 °C. The DTG curves showed that, in the case of the composites, the initial decomposition temperature shifted slightly toward higher values as the TiO_2_ content increased. Although the differences in the thermal stability of the materials were not large, it seems that the best thermal stability was achieved by the polymer composites containing 7 wt.% of samples T and TZ. The residual mass of the polymer composites after the heating process was higher than the filler content of the materials, suggesting that the fillers slightly impeded the diffusion of the gaseous decomposition products (this caused the dry residue mass to be higher). In addition, the composite polymers reinforced with T and TZ had better thermal stability than those with the modified materials T_M_ and TZ_M_. Additionally, slightly lower residual masses were obtained for the materials containing modified fillers than for those containing the same amount of unmodified fillers, which may be due to the thermal decomposition of the silane-modifier fragment.

**Figure 14:**
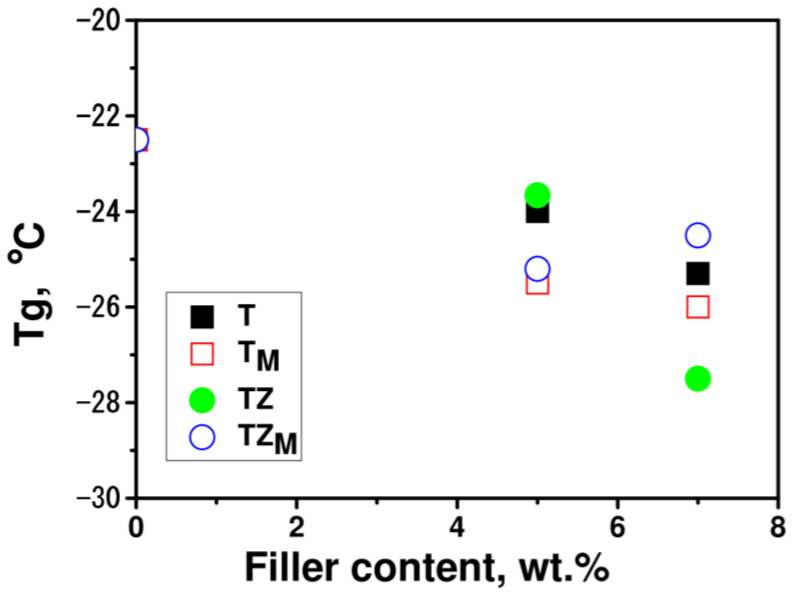
Glass transition temperatures, *T_g_*, of polymer matrix and composites with various types and concentrations of fillers T, T_M_, TZ, and TZ_M_ determined from the first heating cycle.

DSC measurements enabled the determination of glass transition temperatures of the pure polymer matrix polyPEGDA and the prepared composites with various types and concentrations of fillers. The glass transition temperature of pure polyPEGDA was −22.5 °C; thus, the prepared polymer is an elastomer (Figure 14). The addition of synthesized fillers decreased the glass transition temperature of the polymer matrix by approximately 0.5 to 3.0 °C, depending on the type of filler and its concentration in the composite. The silane-modified fillers T_M_ and TZ_M_ exerted a greater effect. The decrease in this parameter may be related to the breaking of interchain bonding in PEGDA by the filler, and hydrophobic silane groups may have a greater influence on this effect.

#### 3.2.5. Morphology of Fabricated Composites

**Figure 15:**
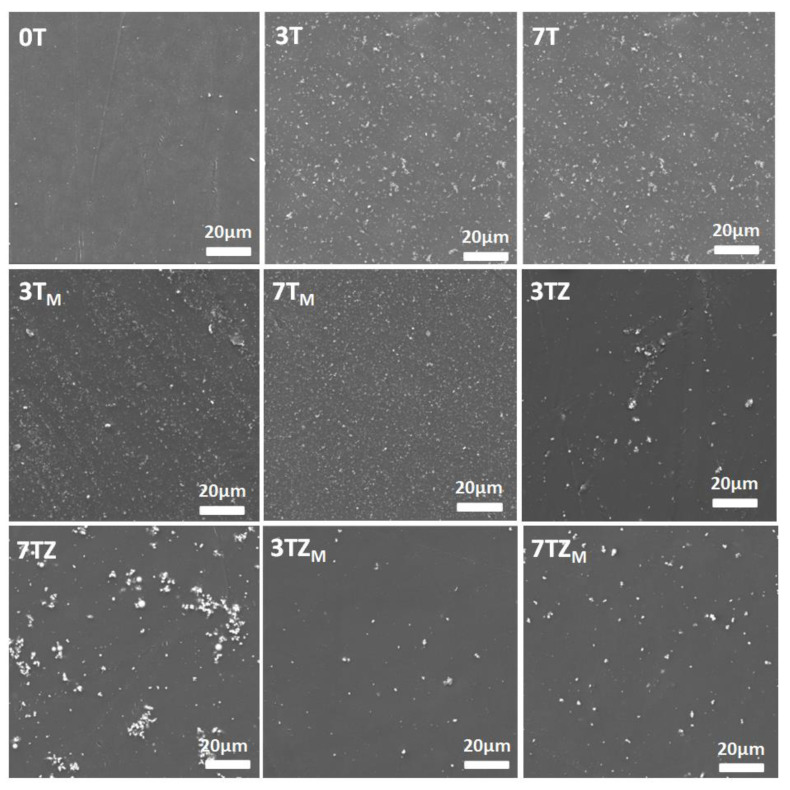
Morphology of the polymer (0) and polymer composites containing inorganic oxide fillers: T, T_M_, TZ, and TZ_M_. The numbers indicate the filler content (in wt.%).

Figure 15 shows SEM images of the surfaces of pure polyacrylate and polymer composites containing the fillers T, T_M_, TZ, and TZ_M_. SEM images of the surface of the unfilled polyPEGDA show a smooth surface with some fine impurities; TiO_2_ particles are represented as tiny white spots on the gray background of the polymer matrix. On the surface of the polymer composite, the particles exhibit angular and spherical shapes. Some of the unmodified particles tend to agglomerate with each other, especially in the composites with higher contents of the unmodified TZ filler. Although the FTIR analysis indicated that there were no strong interfacial interactions between PEGDA and the modified fillers, a uniform distribution of modified TiO_2_ embedded in the polyacrylate matrix was observed, suggesting good interface adhesion and compatibility between the modified particles and polymer matrix, leading to a homogeneous dispersion. Also, the modification of TZ to TZ_M_ samples improved its homogenization in the polymer; although these particles were much smaller and there were far fewer of them on the surface than in the case of T_M_, the aggregates of TZ_M_ were separated and uniformly dispersed in the polymer matrix.

The morphology of the prepared composites seems to confirm the effect of the dispersion of the filler on the photocurable composition on the course of polymerization of the PEGDA monomer. In the cases of T and T_M_ fillers, they are observed to be uniformly dispersed on the surface of the composites, which may block the access of initiating light to the deeper layers of the irradiated composition. However, in the cases of the TZ and TZ_M_ fillers, polymerization is faster and leads to greater conversion, because the filler does not block the penetration of incident light into the composition. The observed increase in *p^f^* in the case of the TZ_M_ filler may indicate its diluting effect on the system, causing an increase in the mobility of the polymer network being formed, or additional effects related to catalytic properties.

#### 3.2.6. Contact Angle

**Figure 16:**
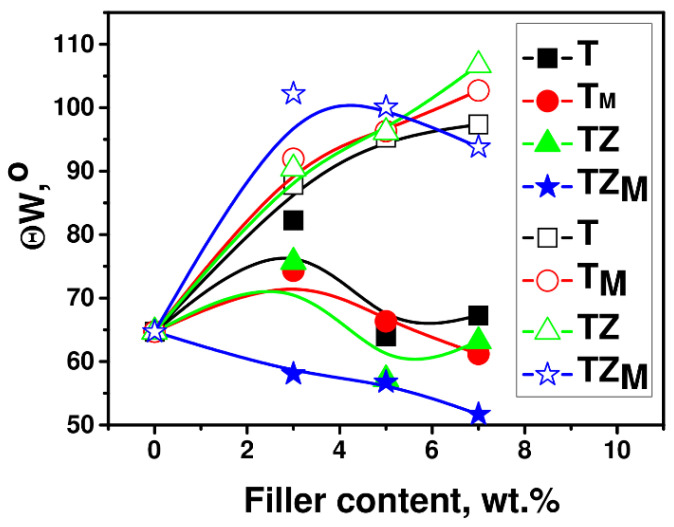
Water contact angle, θW, before (filled symbols) and after (unfilled symbols) UV-curing, as a function of the filler content in polyPEGDA/filler composites containing the fillers T, T_M_, and TZ, TZ_M_.

The hydrophilic or hydrophobic nature of the polymer and composite material surface was assessed by measuring the water contact angle (θW). This parameter was used to detect changes in the hydrophobicity of the polymer matrix. The angle of contact of water as a function of the filler content in polyPEGDA is shown in Figure 16. To determine whether our materials had super-wettability properties (due to the presence of TiO_2_-based systems), the contact angle was measured before and after additional UV-light irradiation (Figure 16). Polymer and composite materials were subjected to additional irradiation for 1.5 h.

**Figure 17:**
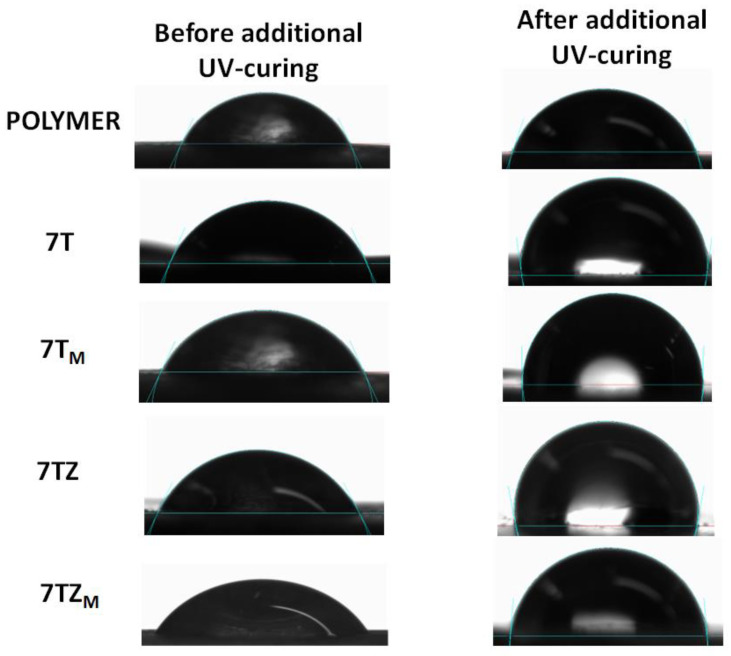
Water contact angle, θW, before and after additional UV curing for polymer and composites containing 7 wt.% of samples T, T_M_ and TZ, TZ_M_.

**Figure 18:**
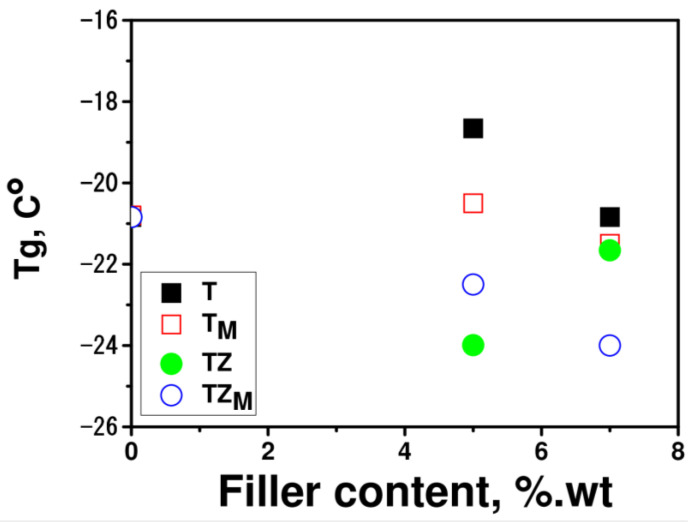
Glass transition temperatures, T_g_, of polymer matrix and composites with various types and concentrations of the fillers T, T_M_, TZ, and TZ_M_, determined from the second heating cycle.

As can be seen from the graphs, the addition of fillers produced only a slight change in the contact angle of the polymer matrix. Except in the system containing TZ_M_, the addition of filler caused a slight increase in or had no effect on the water contact angle, with the effect depending on the filler content. Surprisingly, in light of the current literature data, additional exposure to UV light changed the properties of the polymer matrix, making it more hydrophobic. The hydrophobicity of TiO_2_-based composites improved significantly after additional irradiation with UV light, which increased the contact angle by about 45% (for the samples containing 7 wt.% of filler; Figure 17). Wettability was dependent on the hydrogen bonding network that formed on the surface. An improvement of hydrophilic properties was observed after UV-light irradiation, e.g., in publications [47,48], where the shelf life of dental titanium implant products and properties of orthodontic resin coated with UV-responsive photocatalyst were discussed. Our research was completely different (the light irradiation changed the properties of the prepared coatings to more hydrophobic), indicating that the properties of TiO_2_-based materials could be completely different. The water contact angle increased with the increasing content of filler in the polymer matrix, but interestingly, did not depend on the type of filler. Additional exposure to UV radiation also changed the water contact angle of pure polyPEGDA, which may be related to a higher conversion of the double bonds that were trapped during synthesis in the polymer matrix. Irradiation with such high-intensity UV light also caused an additional effect: an increase in temperature. These two parameters together may have increased the degree of double-bond conversion in the monomer. Thus, the glass transition temperature was also determined from the second heating cycle (Figure 18), to determine whether heating of the sample affected this parameter. In the cases of both the pure polymer matrix and composites, the values of *T_g_* were higher than those obtained from the first heating cycle; *T_g_* was equal to −21 °C for the matrix and lay in a range from −24° to −19 °C for the composites.

As a continuation of the research, it is planned to investigate the influence of TiO_2_-based fillers on the mechanical properties (compressive and tensile strength), depth of curing, and antibacterial properties of a typical dental filling. Since the addition of this filler has such a significant impact on the hydrophilic–hydrophobic properties and degree of conversion of double bonds, extending these tests will enable the modification of the surface properties of the dental composite as well as ionomer cements.

## 4. Conclusions

In this study, surface-modified TiO_2_ and TiO_2_-ZrO_2_ were successfully synthesized using the sol-gel method assisted by a calcination process and subsequent modification with a silane coupling agent, and were used as efficient oxide fillers in a polymer matrix. The synthesis method produced titanium dioxide materials with specific properties. The effectiveness of the method was confirmed by FTIR and SEM analyses. The surface modification introduced functional groups, altering size, surface area, and pore volume. Modified oxide fillers had a lower BET surface area and a larger pore size.

Polymer composites were obtained through photocuring. Photopolymerization was studied using photo-DSC, and the compositions and their components were also characterized using FTIR and UV spectroscopy. Fillers and their modification affected cure kinetics differently. The addition of the fillers reduced the polymerization rate and double-bond conversion, except for the TZ_M_ filler. The modification of TiO_2_ with ZrO_2_ altered the properties of the light-cured composition. Compositions with TZ and TZ_M_ had a low photopolymerization-initiation ability.

The thermal stability of composite materials improves with increasing the filler content, making them suitable for many packaging applications. The hydrophobicity of the TiO_2_-based composite improved with UV irradiation, enhancing possible applications in stomatology. These materials modified with fillers could potentially enhance the properties of polymer-based crowns and denture bases.

## Figures and Tables

**Table 1:** Dispersive properties of inorganic oxide fillers.

Sample	T	T_M_	TZ	TZ_M_
Particle size distribution by volume, nm	164–531	459–1110	342–615	459–825
Maximum volume contribution, %	295 nm–22.7	712 nm–28.2	459 nm–43.0	615 nm–38.8
Polydispersity index, PdI	0.382	0.225	1.000	0.209

**Table 2:** Phase composition of the fabricated inorganic oxide fillers.

Sample		T	T_M_	TZ	TZ_M_
Phase composition (%)	Anatase	100	100	79.2	79.2
	Rutile	-	-	-	-
	ZrTiO_4_	-	-	20.8	20.8
	Monoclinic ZrO_2_	-	-	-	-

**Table 3:** Degree of surface coverage of inorganic oxide systems modified with A-174.

Sample		T	T_M_	TZ	TZ_M_
Elemental analysis (%)	N	0.0254	0.0212	0.0185	0.0228
	C	0.1075	2.5447	0.0933	2.4837
	H	0.0390	0.3469	0.3418	0.4233
P (μmol/m^2^)		-	5.5	-	1.8

**Table 4:** Wavenumbers and functional groups present in the fabricated fillers.

Wavenumber, cm^−1^	Functional Group	Sample				
		A-174	T	T_M_	TZ	TZ_M_
**640**	Ti-O-Ti	−	+	+	+	+
**714**		−	+	+	+	+
**721**	Ti-O-Zr	−	−	−	+	+
**742**	Zr-O	−	−	−	+	+
**830**	Si-CH_2_ (asymmetric)	+	−	+	−	+
**910–890**	Si-OH	+	−	+	−	+
**1080**	Si-O	+	−	+	−	+
**1087**	Si-O-C	+	−	+	−	+
**1160**	Si-O-CH_3_	+	−	+	−	+
**1410**	C-H	+	−	+	−	+
**1170–1080**	Si-O-Ti	−	−	+	−	+
**1640**	O-H	−	+	+	+	+
**1710**	C=O (ester)	+	−	+	−	+
**2830**	-CH_2_- (asymmetric)	+	−	+	−	+
**2930**	C-H	−	+	+	+	+
**2950**	-CH_2_- (symmetric)	+	−	+	−	+
**2973**	-CH_3_	+	−	+	−	+
**3380**	O-H	−	+	+	+	+

+ presence of bonds, − no bands.

**Table 5:** Results from thermal decomposition of the investigated materials: polyPEGDA and its composites with the prepared fillers.

Sample	R (%)	T_5_ (°C)	T_50_ (°C)	T_max_ (°C)
0	4.9	327	395	392
3T	10.5	330	400	397
7T	13.0	337	404	397
3T_M_	5.5	337	397	394
7T_M_	11.2	330	402	397
3TZ	8.87	344	397	394
7TZ	12.7	342	402	394
3TZ_M_	11.3	327	402	399
7TZ_M_	9.84	337	393	400

T_5_ and T_50_ are the temperatures at which the weight loss of a sample reaches 5% and 50%, respectively. R is residual mass. T_max_ is the temperature of the maximum rate of decomposition of the material.

## Data Availability

The original contributions presented in the study are included in the article, further inquiries can be directed to the corresponding author/s.
